# Unraveling the Link: Ferroptosis and Its Implications in Cerebrovascular Diseases

**DOI:** 10.3390/biom16020228

**Published:** 2026-02-02

**Authors:** Zeran Yu, Jiabin Su, Xinjie Gao, Yuchao Fei, Meng Zhang, Junhui Qi, Wei Ni, Yuxiang Gu

**Affiliations:** 1 Department of Neurosurgery, The Affiliated Hospital of Yunnan University, Kunming 650021, China; yuzerancn@gmail.com (Z.Y.);; 2 Department of Neurosurgery, The Second People’s Hospital of Yunnan, Kunming 650021, China; 3 Department of Neurosurgery, Huashan Hospital, Fudan University, Shanghai 200040, China; 4 Neurosurgical Institute, Fudan University, Shanghai 200040, China; 5 Shanghai Clinical Medical Center of Neurosurgery, Shanghai 200040, China; 6 National Center for Neurological Disorders, Huashan Hospital, Shanghai Medical College, Fudan University, Shanghai 200040, China; 7 Department of Neurosurgery, Liaocheng Brain Hospital, The People’s Hospital of Liaocheng, Liaocheng 252000, China; 8 Department of Neurosurgery, The First Affiliated Hospital of Fujian Medical University, Fuzhou 350005, China

**Keywords:** ferroptosis, intracerebral hemorrhage, intracranial aneurysms, ischemic stroke, subarachnoid hemorrhage, atherosclerosis

## Abstract

Cerebrovascular diseases, encompassing a spectrum of conditions affecting the blood vessels supplying the brain, represent a significant global health burden. Among the diverse mechanisms implicated in cerebrovascular pathology, emerging evidence highlights the role of ferroptosis, a regulated form of cell death characterized by iron-dependent lipid peroxidation. The review also elucidates the molecular mechanisms underlying ferroptosis, emphasizing the pivotal role of iron, the intracellular antioxidant system, and lipid metabolism. Subsequently, it explores the growing body of literature implicating ferroptosis in the pathogenesis of various cerebrovascular diseases, including atherosclerosis, ischemic stroke, intracerebral hemorrhage, and subarachnoid hemorrhage. Special attention is given to the interplay between ferroptosis and other established mechanisms, such as oxidative stress, and inflammation. Moreover, pharmacological interventions and therapeutic strategies aimed at modulating key players in the ferroptosis cascade are explored, with a focus on their translational potential for clinical application. Finally, the review addresses current gaps in knowledge and proposes future research directions, emphasizing the need for a deeper understanding of the specific roles of ferroptosis in the pathogenesis of cerebrovascular diseases. The elucidation of these aspects holds promise for advancing our comprehension of cerebrovascular pathology and opening new avenues for therapeutic intervention in these debilitating conditions.

## 1. Introduction

Ferroptosis is a recently recognized form of iron-dependent regulated cell death that is mechanistically and morphologically distinct from other programmed cell death modalities, including apoptosis and necroptosis [1]. It is characterized by mitochondrial shrinkage, loss of mitochondrial cristae, and increased mitochondrial membrane density, while the plasma membrane and nucleus largely remain intact [2]. Accumulating evidence indicates that ferroptosis contributes to the pathogenesis of a broad spectrum of diseases across multiple organ systems. Within the central nervous system, ferroptosis has been increasingly implicated in neurodegenerative disorders [3,4], traumatic brain injury [5], and other forms of neurological damage [6]. Cerebrovascular diseases, encompassing both hemorrhagic and ischemic stroke, represent a major cause of mortality and long-term disability worldwide [7]. In this context, growing attention has been directed toward the potential involvement of ferroptosis in cerebrovascular injury. Accordingly, this review summarizes current knowledge regarding the metabolic mechanisms underlying ferroptosis and highlights its emerging roles in cerebrovascular diseases.

## 2. The Cellular Metabolic Mechanisms of Ferroptosis

### 2.1. Metabolism of Iron

**Figure 1:**
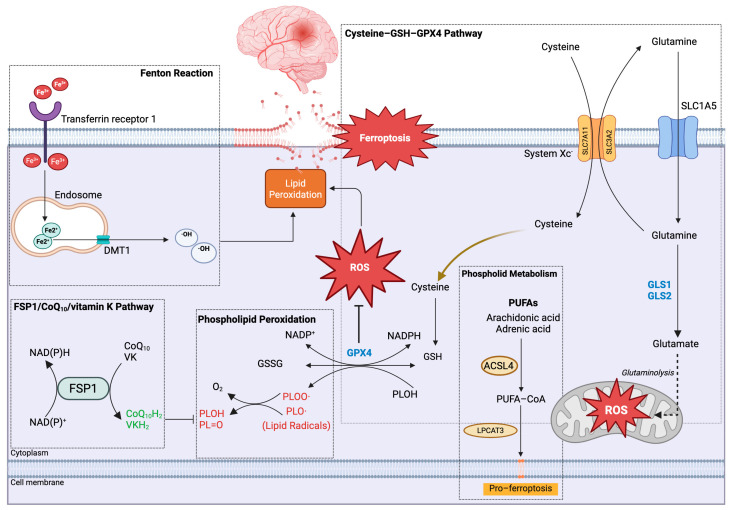
The mechanism of ferroptosis involves a sequence of intricate molecular events. Ferric iron (Fe^3+^) binds to transferrin (TF) and is internalized into cells via transferrin receptor 1 (TFR1). Within endosomes, Fe^3+^ is reduced to ferrous iron (Fe^2+^) by six-transmembrane epithelial antigen of the prostate 3 (STEAP3), followed by Fe^2+^ export into the cytosol through divalent metal transporter 1 (DMT1). Excess cytosolic Fe^2+^ promotes reactive oxygen species (ROS) generation through the Fenton reaction and iron-catalyzed enzymatic processes, facilitating lipoxygenase (LOX) activation and subsequent oxidation of polyunsaturated fatty acids (PUFAs), ultimately driving ferroptosis. In parallel, system Xc^−^ mediates cystine uptake in exchange for glutamate export. Intracellular cystine is reduced to cysteine, supporting glutathione (GSH) synthesis. GSH acts as an essential reducing cofactor for glutathione peroxidase 4 (GPX4), enabling the enzymatic reduction in phospholipid hydroperoxides derived from polyunsaturated fatty acids (PUFA-OOH) into their corresponding non-toxic lipid alcohols (PUFA-OH), thereby preventing lipid peroxidation propagation and suppressing ferroptosis. Additionally, ferroptosis suppressor protein 1 (FSP1) functions at the plasma membrane as an NAD(P)H-dependent oxidoreductase to regenerate reduced coenzyme Q10 (ubiquinol), scavenging lipid peroxyl radicals and limiting lipid peroxidation independently of GPX4. Perturbations in amino acid metabolism and excessive lipid peroxide accumulation collectively contribute to the induction of ferroptosis.

As implied by the term, iron assumes a pivotal role in the process of cell death in ferroptosis. Upon absorption from the gastrointestinal tract through dietary intake, iron in the bloodstream promptly associates with the iron-transporting protein transferrin (Tf). Transferrin serves as a carrier, facilitating the transportation of iron to various tissues and cells. The cell surface hosts transferrin receptor 1, which binds with transferrin, forming a complex. This complex undergoes clathrin-mediated endocytosis, transforming into an endosome. Ultimately, the iron contained within the endosome is conveyed into the cellular plasma through the action of the divalent metal transporter (DMT1) [8]. Iron plays a vital role in cellular energy acquisition through oxidative phosphorylation occurring at the mitochondrial cristae, leading to the production of adenosine triphosphate (ATP). Upon ingress into the mitochondria, iron engages with sulfur, giving rise to iron–sulfur clusters and heme groups within the proteins constituting the electron transport chain. This interaction facilitates the generation of a proton gradient, enabling ATP synthase to catalyze the synthesis of ATP [9]. Excess iron in cells can be excreted as ion efflux by ferroportin 1 (fpn1) on the cell membrane [10]. Upon the accumulation of iron within cellular compartments, intracellular ferrous ions (Fe^2+^) undergo reactions with hydrogen peroxide, giving rise to potent oxidizing agents, notably hydroxyl radicals (OH·), hydroxide ions (OH^−^), and ferric ions (Fe^3+^) [11]. These reactive oxygen species (ROS) possess the propensity to interact with cellular membrane lipids, thereby instigating lipid peroxidation (Figure 1).

### 2.2. Metabolism of Lipid Peroxide

The primary constituents of cellular membrane phospholipid bilayers are polyunsaturated fatty acids (PUFAs), including linoleic acid, arachidonic acid, and docosahexaenoic acid [12]. PUFAs are particularly susceptible to peroxidation. Additionally, the acyl-CoA synthetase long-chain family 4 (ACSL4) plays a pivotal role in various lipid-related processes such as fatty acid synthesis, β-oxidation, and alkyl lipid synthesis (Figure 1). ACSL4 facilitates the conversion of PUFAs into fatty acyl-CoA, subsequently leading to esterification through lysophosphatidylcholine acyltransferase 3 (LPCAT3), resulting in the generation of phospholipids [13]. In pathological conditions like inflammation, malignancy, and trauma, increased levels of reactive oxygen species (ROS) are produced through the catalytic activity of ALOX15 on intracellular free phospholipids. These ROS exhibit robust oxidative potential and readily react with cellular lipids, proteins, and DNA [14]. Under such circumstances, a substantial quantity of generated ROS reacts with the vulnerable double bond structure of PUFAs, leading to the formation of lipid peroxidation free radicals. This cascade of events exacerbates lipid peroxidation, ultimately culminating in cell membrane rupture and ferroptosis [13].

### 2.3. Regulation of Ferroptosis by the System Xc^−^-GSH-GPX4 Antioxidant Axis

Reactive oxygen species (ROS) are integral to the ferroptotic process (Figure 1). To shield cells from ROS and consequent peroxidation, an intracellular antioxidative system operates. System Xc^−^ functions as an amino acid antiporter, comprising a light chain (SLC7A11) and a heavy chain (SLC3A2) on the cell membrane. This system transports one glutamate out of the cell while simultaneously importing one cystine. Once inside the cell, cystine undergoes prompt reduction facilitated by cystine reductase, transforming it into cysteine—an essential precursor for the synthesis of the antioxidant glutathione (GSH) [15]. Subsequently, GSH assumes a pivotal role as a substrate for the antioxidant enzyme glutathione peroxidase 4 (GPX4), actively participating in the reduction of lipid peroxides and their derivatives [16]. This process effectively safeguards cells against oxidative damage.

### 2.4. Context-Dependent Regulation of Ferroptosis by p53

Furthermore, p53 also plays a role in ferroptosis. Since p53 was discovered in the late 1970s, its function in tumor growth has been extensively studied. It is generally believed that p53 could be activated to limit potential damage from gene mutation after DNA injury by inhibiting the cell cycle and promoting cell self-repair [17]. ROS could induce the expression of p53, but p53 might act entirely differently in various contexts. Under normal conditions, p53 promotes antioxidant activity, whereas under oxidative stress, p53 raises pro-oxidant activity [18]. In addition, p53 is also involved in the glutathione system. SLC7A11 encoding system Xc^−^ is one of the target genes of p53. Activated p53 confines both the expression and function of SLC7A11, limiting glutathione production and inducing ferroptosis [19]. However, another study focusing on vascular wall calcification in hyperlipidemia found that p53 activation upregulates the expression level of SLC7A11 and glutathione [20]. The abovementioned results may indicate that the effects of p53 could vary in different research conditions [17]. In addition, p53 also involves other metabolic pathways in ferroptosis. It is well-established that GPX4 inhibition induces ferroptosis. In p53-mediated ferroptosis, however, GPX4 function remains unchanged. Moreover, ferroptosis induced by GPX4 inhibition requires the involvement of ACSL4, which is not required in p53-mediated ferroptosis. In addition, ALOX12 is crucial for p53-mediated ferroptosis but not in GPX4-inhibited ferroptosis [21].

## 3. Ferroptosis Detection in Neurovascular Disease

A fundamental challenge in ferroptosis research is the lack of well-established, specific markers that allow for the direct detection of ferroptotic cell death. Consequently, the contribution of ferroptosis to tissue injury is typically inferred using a combination of complementary experimental approaches rather than a single definitive assay. Current methodologies generally rely on four key criteria: (1) confirmation of cell death, (2) rescue of cell death by ferroptosis-specific inhibitors, (3) detection of lipid peroxidation, and (4) assessment of ferroptosis-related gene and protein expression changes [22].

### 3.1. Detection of Cell Death

In vitro, cell death is commonly assessed using conventional approaches such as cell viability assays, lactate dehydrogenase (LDH) release, classical cell death staining, and morphological assessment [23]. In vivo, cell death is typically examined by transmission electron microscopy to identify characteristic ultrastructural changes, as well as by terminal deoxynucleotidyl transferase dUTP nick-end labeling (TUNEL) staining, which detects DNA strand breaks in tissue sections. Although TUNEL was initially regarded as a specific marker of apoptosis, it is now recognized to label multiple forms of regulated cell death, including ferroptosis [24]. Notably, in studies of the neurovascular unit, TUNEL-based detection may be technically challenging because cell death signals are often subtle and spatially restricted, thereby limiting sensitivity and interpretability in cerebrovascular disease models [25].

### 3.2. Detection of Lipid Peroxidation

Lipid peroxidation represents a central biochemical hallmark of ferroptosis and thus represents a critical parameter for its identification. In vitro, lipid peroxidation is commonly assessed using fluorescent probes such as BODIPY 581/591 C11 or STY-BODIPY. However, these probes are generally unsuitable for direct tissue staining or in vivo applications, unless freshly isolated cells from tissue samples are used [26]. As an alternative, liquid chromatography–tandem mass spectrometry (LC–MS/MS) enables comprehensive profiling of oxidized lipid species and provides a more detailed assessment of lipid peroxidation status [27]. Despite these advances, major challenges remain. There is currently no consensus regarding which specific phospholipid species, or what threshold of lipid oxidation, definitively defines ferroptosis [28]. Moreover, lipid peroxidation is not unique to ferroptosis, as modest increases may also occur in other forms of regulated cell death or generalized oxidative injury [29]. Therefore, measurements of lipid peroxidation alone are insufficient to establish ferroptosis and should be interpreted in combination with functional rescue experiments and ferroptosis-related molecular signatures.

### 3.3. Detection of Ferroptosis-Related Protein

Glutathione peroxidase 4 (GPX4), SLC7A11, prostaglandin-endoperoxide synthase 2 (PTGS2), and acyl-CoA synthetase long-chain family member 4 (ACSL4) are among the most widely studied proteins involved in the regulation of ferroptosis. Changes in the expression of these key regulators can be assessed using immunodetection techniques, including immunoblotting, immunofluorescence (IF), and immunohistochemistry (IHC), which enable both quantitative and spatial evaluation of ferroptosis-related proteins in cells and tissues.

GPX4 plays a central protective role against ferroptosis by reducing phospholipid hydroperoxides to non-toxic lipid alcohols in a glutathione (GSH)–dependent manner [30]. Accordingly, decreased GPX4 expression or impaired enzymatic activity is a critical trigger for ferroptotic cell death. Beyond measuring GPX4 protein levels, its functional activity can be further evaluated by assessing GSH-dependent phospholipid hydroperoxide reduction [31]. Moreover, cell-type–specific expression of ferroptosis-related proteins within brain tissue can be resolved by IF co-localization approaches [32,33]. Notably, in models of cerebrovascular disease, while neuronal ferroptosis has been well documented, whether glial populations such as microglia and astrocytes also undergo ferroptosis, and how their interactions influence neuronal injury, remain important questions for future investigation.

### 3.4. Neuroimaging Detection of Iron

Iron, particularly in its ferric (Fe^3+^) form, exhibits strong paramagnetic properties that induce local magnetic field inhomogeneities by accelerating the nuclear spin relaxation of surrounding water protons. This effect leads to shortening of T2 and T2* relaxation times and a corresponding reduction in magnetic resonance (MR) signal intensity [34]. Consequently, iron-rich regions demonstrate characteristic signal changes across conventional MRI sequences. Under standard clinical parameters, areas with iron deposition typically appear hypointense on T2-weighted images and isointense on T1-weighted images, while T2*-weighted gradient-echo acquisitions further accentuate signal loss through a “blooming” effect. Susceptibility-weighted imaging (SWI) is particularly sensitive to magnetic susceptibility differences and markedly enhances hypointense signals associated with iron accumulation. Notably, the magnetic effects of iron depend on its oxidation state, as biologically relevant iron oxides containing Fe^2+^ possess fewer unpaired electrons than Fe^3+^ and are therefore less effective in quenching T2-weighted signal intensity [35]. Quantitative susceptibility mapping (QSM) has emerged as an advanced post-processing technique that reconstructs the spatial distribution of tissue magnetic susceptibility in vivo from gradient-echo magnetic resonance phase images [36]. Because magnetic susceptibility in gray matter exhibits a strong linear correlation with chemically measured iron concentration, QSM provides a robust, non-invasive approach for quantifying pathological iron accumulation in the human brain [37,38]. Accordingly, susceptibility values derived from QSM are increasingly recognized as potential imaging biomarkers for cerebrovascular diseases.

Recent studies have applied QSM to characterize regional brain iron deposition and its clinical relevance. In patients with cerebral small vessel disease (CSVD), 3.0T MRI-based QSM revealed significantly elevated susceptibility values in the right Rolandic operculum and right superior temporal gyrus, indicating increased iron accumulation that was directly associated with impairments in executive function and memory [39]. In the context of acute ischemic stroke (AIS), QSM analyses have demonstrated increased iron concentrations in the caudate nucleus during the acute phase. Conversely, reduced iron content in the contralateral caudate nucleus has been associated with poorer functional outcomes in AIS patients [40]. Beyond parenchymal iron deposition, emerging evidence indicates that QSM may also be capable of detecting intraplaque hemorrhage within atherosclerotic lesions, further expanding its utility in cerebrovascular disease assessment [41]. Collectively, these findings underscore the value of QSM as a non-invasive imaging tool for revealing the spatial heterogeneity of iron dysregulation and its prognostic relevance across a spectrum of cerebrovascular pathologies.

## 4. Ferroptosis in Intracerebral Hemorrhage

Intracerebral hemorrhage (ICH), characterized by the rupture of cerebral vessels and subsequent blood extravasation into the brain parenchyma, accounts for approximately 10–15% of all stroke cases and is associated with high mortality and disability rates [42,43]. Increasing evidence indicates that ferroptosis, an iron-dependent form of regulated cell death driven by lipid peroxidation, plays a pivotal role in secondary neuronal injury following ICH.

Following hemorrhage, erythrocyte lysis leads to the accumulation of hemoglobin degradation products, particularly heme. Under the action of heme oxygenase, heme is metabolized into iron, carbon monoxide, and biliverdin, all of which contribute to oxidative stress, neuroinflammation, and lipid peroxidation within the perihematomal brain tissue [44]. Excess iron acts as a central catalyst in ferroptosis by promoting reactive oxygen species (ROS) generation and membrane lipid oxidation, thereby accelerating neuronal death [45]. Experimental studies using in vitro ICH models suggest that ferroptosis may account for more than 80% of total neuronal death, underscoring its dominant contribution to hemorrhage-induced neurotoxicity [46]. Notably, ferroptotic neuronal injury is not confined to regions directly adjacent to the hematoma but can also be detected in anatomically distant areas, indicating a diffuse and progressive pathological process [47].

ICH also profoundly disrupts neuronal redox homeostasis and mitochondrial function. The expression of NADPH oxidase 4 (NOX4) is significantly upregulated in neurons following ICH, resulting in impaired excitatory amino acid transporter 3 (EAAT3) activity and reduced glutathione synthesis, thereby weakening intrinsic antioxidant defenses [48]. In parallel, NOX4-mediated electron transfer from the mitochondrial membrane to the cytoplasm enhances ROS production and facilitates ferroptotic signaling [49,50]. Mitochondrial dysfunction further amplifies ferroptosis, as inhibition of electron transport chain complex I exacerbates oxidative stress and lipid peroxidation in neurons after ICH [51]. Systemic metabolic disturbances, such as hyperglycemia, may additionally aggravate ferroptosis by suppressing PPAR-γ activity in neutrophils, leading to decreased lactoferrin expression, enhanced neuronal iron accumulation, and subsequent ferroptotic cell death [52].

Given the central role of ferroptosis in post-ICH neuronal injury, substantial efforts have been devoted to exploring anti-ferroptotic therapeutic strategies. One major approach involves limiting iron availability and utilization. Dexmedetomidine, an α2-adrenergic agonist commonly used in neurocritical care, has been shown to downregulate transferrin receptor 1 (Tfr1) and upregulate GPX4 expression in experimental ICH models, thereby attenuating neuronal ferroptosis and improving neurological outcomes [53]. However, whether these protective effects translate into clinical benefit for ICH patients remains to be determined. Additionally, iron accumulation after ICH may activate hypoxia-inducible factor prolyl hydroxylase domain (HIF-PHD), triggering ATF4-dependent neuronal death pathways, further linking iron metabolism to ferroptotic injury [54].

Another promising strategy focuses on enhancing antioxidant capacity. Neurons may counteract ferroptosis by upregulating selenoprotein synthesis, particularly glutathione peroxidase 4 (GPX4). Selenium supplementation has been shown to activate transcription factors such as TFAP2c and Sp1, thereby promoting GPX4 expression and conferring neuroprotection after ICH [55]. On this basis, selenium-containing compounds with improved blood–brain barrier permeability have been developed and demonstrated efficacy in mitigating ferroptosis in experimental ICH models [56,57]. In addition, the long non-coding RNA HOTAIR has been identified as a critical mediator of the neuroprotective effects of paeonol, suppressing ACSL4 expression and preventing UPF1 degradation, ultimately inhibiting neuronal ferroptosis and improving post-ICH outcomes [58]. Crocin, a bioactive compound derived from saffron, alleviates oxidative stress and ferroptosis by promoting Nrf2 nuclear translocation, thereby reducing neuronal damage following ICH [59].

Furthermore, modulation of neuroinflammation through microglial regulation has emerged as an indirect yet important strategy. Artesunate induces ferroptosis selectively in pro-inflammatory M1-polarized microglia via the AMPK–mTOR–GPX4 pathway, thereby attenuating secondary neuronal injury mediated by inflammatory cytokines and improving neurological function after ICH [49]. Similarly, the DPP-4 inhibitor vildagliptin suppresses microglial activation and reduces neuronal iron accumulation, contributing to ferroptosis inhibition and neuroprotection [60].

Despite these advances, several mechanistic questions remain unresolved. For instance, ICH has been reported to activate sphingosine kinase 1 (Sphk1), subsequently enhancing ERK/p-ERK signaling and accelerating neuronal ferroptosis [61]. However, the upstream triggers of Sphk1 activation following hemorrhage and the feasibility of pharmacologically targeting this pathway in ICH require further investigation.

## 5. Ferroptosis in Atherosclerosis

Atherosclerosis is a persistent inflammatory ailment primarily affecting the carotid artery and cerebral blood vessels, intimately linked with intracranial artery stenosis, ischemic stroke, and intracranial aneurysms [62,63]. Typically, endothelial cells within blood vessels are intricately connected through intracellular junctions. Under the shear stress exerted by blood flow, lipoproteins within the bloodstream can permeate the intimal layer of the blood vessel wall. Following this, endothelial cells express receptors that recruit peripheral monocytes, prompting their migration into the intima, where they differentiate into macrophages. These macrophages subsequently uptake lipoproteins, transforming into foam cells [64]. As the disease advances, foam cells gradually evolve into cholesterol-rich necrotic cores, prone to rupture, attracting platelet aggregation, and culminating in thrombotic occlusion, ultimately leading to brain ischemia. Historically, apoptosis was regarded as one of the characteristic features of atherosclerosis [65,66].

In recent years, a growing body of evidence has illuminated the involvement of ferroptosis in atherosclerosis. Human atherosclerotic lesions are identified for free iron through the bleomycin assay and electron paramagnetic resonance spectroscopy [67,68]. There are several sources of iron in atherosclerotic lesions. First, iron arises from the lysis of erythrocytes, either leaking out from the rupture of fragile small vessels beneath the plaques or squeezing into atherosclerotic lesions through fissures in the plaque cap [69,70,71]. Myeloperoxidase (MPO) serves as an additional source of iron. Following erythrocyte entry into the plaque, recruited peripheral neutrophils and macrophages phagocytize erythrocytes, with MPO catalyzing the formation of hypochlorous acid—a potent oxidant that induces iron release [72,73] and weakens plaque caps [74]. Furthermore, mechanical injury to the blood vessel wall results in cell death and the subsequent release of catalytic iron from intracellular iron pools within atherosclerotic lesions [75]. Additionally, the degradation of ferritin, cytochrome c, and heme also contributes to the accumulation of iron in atherosclerotic plaques [76,77,78].

Since 1981, the “iron hypothesis” has been posited to elucidate sex differences in cardiovascular disease, particularly the heightened incidence observed in menopausal women [79]. Numerous human and animal studies have identified correlated relationships between iron levels and the severity of the disease [80,81,82]. Notably, non–transferrin-bound iron has been shown to accumulate within the aortic media, creating a highly oxidative and pro-apoptotic vascular microenvironment that promotes plaque instability [80]. These observations provide a mechanistic basis for investigating ferroptosis as a contributor to atherosclerotic progression.

Direct experimental evidence linking ferroptosis to atherosclerosis has emerged from studies employing classical ferroptosis inhibitors. Ferrostatin-1 (Fer-1) has been shown to suppress endothelial cell death and attenuate atherosclerotic lesion development, supporting a functional role of ferroptosis in disease pathogenesis [83]. In a *JAK2^V617F^* overexpression mouse model, increased red cell distribution width was associated with enhanced iron deposition within plaques, accompanied by marked lipid peroxidation and ferroptotic features. In this context, the ferroptosis inhibitor liproxstatin-1 reduced macrophage phagocytosis of erythrocytes, alleviated endothelial injury, and significantly limited atherosclerotic plaque formation [84]. Importantly, the protective effects of Fer-1 appear to be cell-type specific: while effective in endothelial cells, Fer-1 fails to rescue vascular smooth muscle cells from ferroptotic injury in vitro, underscoring heterogeneity in ferroptosis sensitivity within the vascular wall [85].

Dysregulation of antioxidant defense systems further amplifies ferroptosis during atherosclerosis. N-acetylneuraminic acid promotes ubiquitination and p62-mediated degradation of SLC7A11, thereby impairing the SLC7A11/GSH/GPX4 antioxidant axis in endothelial cells and exacerbating ferroptosis and plaque development [86]. Metabolic stress also plays a critical role, as high-fat diet–induced diabetes elevates heme oxygenase-1 expression, suppresses SLC7A11 and GPX4, and triggers ferroptosis in diabetic atherosclerosis [87]. In parallel, cigarette tar stimulates macrophage-derived hepcidin release, leading to intracellular iron accumulation, inhibition of SLC7A11, depletion of glutathione, and enhanced lipid peroxidation [88].

Notably, ferroptosis-related molecular signatures exhibit stage-dependent changes during atherosclerotic progression. In early lesions, GPX4 expression is relatively elevated, whereas ACSL4 and PTGS2 levels remain low or unchanged. In contrast, advanced plaques are characterized by reduced GPX4 expression and marked upregulation of ACSL4 and PTGS2, reflecting a shift toward a ferroptosis-prone state [89]. However, current studies often lack detailed stratification of plaques by pathological stage, limiting mechanistic resolution. This gap highlights the need for future investigations to systematically analyze iron deposition patterns and ferroptosis-related protein dynamics across different stages of atherosclerosis, which may ultimately guide stage-specific anti-ferroptotic therapeutic strategies.

## 6. Ferroptosis in Ischemic Stroke

Ischemic stroke arises from a diminished or complete obstruction of blood flow within intracranial arteries, constituting approximately 80% of all strokes [90]. Intracranial atherosclerotic stenosis stands out as one of the predominant causes of ischemic stroke globally, correlating with significant morbidity and mortality rates [91,92]. Beyond the previously discussed evidence implicating ferroptosis in the pathogenesis of cerebrovascular atherosclerosis, it is noteworthy that ferroptosis also plays a contributory role in the neural injury occurring as a secondary consequence of brain infarction after arterial stenosis. Prior to the introduction of the concept of ferroptosis, elevated iron accumulation in the basal ganglia following ischemic stroke had been documented [93]. Clinical investigations have highlighted a correlation between serum ferritin levels and unfavorable outcomes in acute ischemic stroke cases [94]. Although it is theorized that ischemic events compromise the integrity of the blood–brain barrier, allowing serum iron to leak into the brain [95], the specific mechanism governing iron entry into the ischemic region of the brain remains an area requiring further exploration. Damage to the blood–brain barrier is postulated as a potential instigator of hemorrhagic transformation after brain ischemia [96], a phenomenon associated with ferroptosis. In this context, the P2RX7 receptor, identified as an ATP-gated, non-selective cation channel with widespread expression in astrocytes, microglia, and brain capillary endothelial cells, assumes significance. Notably, hyperglycemia enhances the expression of P2RX7 through the ERK1/2 and p53 signaling pathways within the ischemic brain area and vascular endothelia. Following brain ischemia, the ERK1/2 and p53 signaling pathways mediate ferroptosis of the vascular endothelium [97]. This process compromises the integrity of the blood–brain barrier, expediting hemorrhagic transformation [84]. Furthermore, ischemic neurons instigate USP14 to deubiquitinate NCOA4, initiating ferritinophagy. This cellular mechanism involves the lysosomal digestion of iron-laden ferritin, releasing iron and ultimately leading to neuronal ferroptosis [98].

PUFAs are abundant in the brain, rendering it more susceptible to lipid peroxidation during ischemic events [95]. While the precise mechanism of lipid peroxidation in ischemic stroke remains unclear, substantial evidence substantiates its involvement in brain ischemic injury [99,100,101]. 4-hydroxynonenal (4-HNE), a lipid peroxidation–derived biomarker of ferroptosis, shows potential for predicting clinical outcomes in cerebral ischemia. A case–control study in acute ischemic stroke patients undergoing endovascular thrombectomy demonstrated elevated plasma 4-HNE levels before and after the procedure, which were associated with lower preoperative ASPECT scores and larger infarct core volumes, supporting a link between ferroptosis-related lipid peroxidation and ischemic brain injury severity [102,103].

Various strategies can be employed to safeguard the brain against damage induced by ischemia-induced ferroptosis. One such approach involves synaptosomal-associated protein 25 (SNAP25), which forms a stable complex with syntaxin and synaptobrevin and is prominently expressed in the neuronal plasma membrane and synaptic vesicles. SNAP25 plays a pivotal role in vesicle trafficking, neurotransmitter release, and neuronal plasticity [104]. In response to escalating levels of reactive oxygen species (ROS) following ischemia, the brain increases the expression of SNAP25 [105]. Moreover, ischemic conditions induce an elevation in the expression of ELAVL1 in the brain, contributing to a reduction in the volume of brain infarction in rats. ELAVL1 achieves this effect by stabilizing *DNMT3B* mRNA and inhibiting DNMT3B-mediated methylation of PINK1 [106]. Additionally, the infarcted brain produces a substantial amount of Elabela and its apelin receptor to counteract lipid peroxidation and ferroptosis in neurons through the NRF2/ARE signaling pathway [107]. Although ferrostatin-1 (Fer-1) is a potent inhibitor of ferroptosis in vitro, its application in vivo is limited by poor metabolic stability, which restricts its utility in ferroptosis rescue studies [28]. To address these limitations, considerable efforts have been made to improve its bioavailability and blood–brain barrier (BBB) permeability. Among these strategies, the development of the Fer-1 analog Ferfluor-1 represents a notable advance, as it exhibits enhanced BBB penetration and effectively attenuates neuronal ferroptosis in mouse models of middle cerebral artery occlusion (MCAO) [108]. In parallel, nanotechnology-based delivery approaches have been explored, with lipid nanocarriers such as transferrin-modified lipid nanoparticles (T-LNPs) encapsulating Fer-1 shown to significantly enhance BBB transport, reduce cerebral infarct volume, and potentiate neuroprotective effects in experimental stroke models [22]. Rosmarinic acid encapsulated within nanoliposomes effectively enhances blood–brain barrier permeability and alleviates neuronal ferroptosis after ischemic stroke by binding to KEAP1 and stabilizing NRF2 [108].

Additionally, both traditional Chinese medicine and synthetic compounds exhibit therapeutic potential in alleviating ferroptosis associated with ischemic stroke. Rhein, a natural anthraquinone compound derived from the traditional Chinese herb rhubarb, demonstrates protective effects against ischemic-perfusion injury in HT-22 mouse hippocampal neuronal cells. This protection is achieved through chemical interactions with NRF2, subsequently modulating the expression of crucial factors in the NRF2/SLC7A11/GPX4 pathways implicated in ferroptosis [109]. Icariside II also exhibits a direct binding capability to NRF2, enhancing its transcription and activating the OXPHOX/NF-κB/ferroptosis axis [110]. *Salvia miltiorrhiza* (Danshen) and *Flos Carthami* (Honghua), active components in Danhong injection, widely employed in cardiovascular and cerebrovascular disease treatment in China, have notable effects on cerebral ischemia [111]. Danhong injection enhances the expression of *SATB1*, *SLC7A11*, and *HO-1*, promoting cellular reductant production and diminishing the accumulation of lipid peroxides and ferroptosis in neurons by elevating SATB1 levels [112]. Extracted from the root of the traditional Chinese herb Salvia Miltiorrhiza bunge, 15, 16-Dihydrotanshinone I safeguards mouse brains from ischemic injury by activating NRF2 signaling and inhibiting ferroptosis [113]. β-caryophyllene, a natural compound found in various plants like pepper, blackcurrant, and nutmeg [114,115,116], mitigates ROS and iron accumulation by activating Nrf2 to upregulate HO-1 levels in neurons [117]. Additionally, two phenothiazine derivatives exert preventive effects against erastin-induced ferroptosis through their antioxidant properties [118].

## 7. Ferroptosis in Intracranial Aneurysm and Subarachnoid Hemorrhage

Intracranial aneurysm (IA) and subarachnoid hemorrhage (SAH) represent two closely interconnected stages along a continuous spectrum of hemorrhagic cerebrovascular disease. IA is the leading cause of non-traumatic SAH, with aneurysmal rupture accounting for the majority of spontaneous SAH cases [119,120]. Rather than being independent pathological entities, IA and SAH are linked through a continuous cascade of vascular wall degeneration, hemodynamic stress, and blood-brain interface disruption. Viewing IA and SAH as pathophysiologically unified conditions provides an integrated framework for understanding disease initiation and rupture, and highlights ferroptosis as a potential shared mechanism and therapeutic target across this continuum.

Accumulating evidence suggests that ferroptosis may play a critical role in the formation, progression, and rupture risk of IA. However, mechanistic studies in IA are challenged by intrinsic limitations, including the thin aneurysmal wall, limited tissue availability, and the predominance of minimally invasive endovascular interventions, which restrict access to surgical specimens. Consequently, current investigations largely rely on multimodal approaches combining advanced neuroimaging, histopathological analysis, bioinformatics, and circulating metabolic profiling to infer pathogenic mechanisms (Table 1).

High-resolution susceptibility-weighted imaging (SWI) has provided in vivo evidence of iron accumulation within aneurysm walls. Using 7-T MRI, pronounced hypointense signals consistent with hemosiderin deposition have been observed in giant intracranial aneurysms, with subsequent histopathological analysis confirming iron deposition between the smooth muscle layer and the adventitia [121]. In patients with SAH and initially negative CT angiography, combined MR-SWI and digital subtraction angiography revealed focal, thick, semi-circumferential hypointense signals along the vessel wall of basilar artery perforators, suggestive of hemosiderin accumulation and a dissecting origin of these aneurysms [122]. Importantly, iron deposition within the aneurysm wall has been shown to correlate with increased expression of cyclooxygenase-2 (COX-2), a recognized ferroptosis-associated marker. Elevated COX-2 expression is further associated with aneurysm instability and an increased likelihood of future rupture [123]. Beyond imaging and histological observations, emerging molecular studies reinforce the link between ferroptosis and IA. Bioinformatic analyses have identified ferroptosis-related genes and competitive endogenous RNA networks associated with aneurysm formation and progression [124,125]. Complementing these findings, our prior clinical cohort study demonstrated elevated serum glutamine levels—a key metabolite involved in ferroptosis regulation—in patients harboring unstable intracranial aneurysms [126]. These data collectively suggest that metabolic reprogramming and ferroptosis-related pathways may contribute to aneurysm vulnerability.

**Figure 2:**
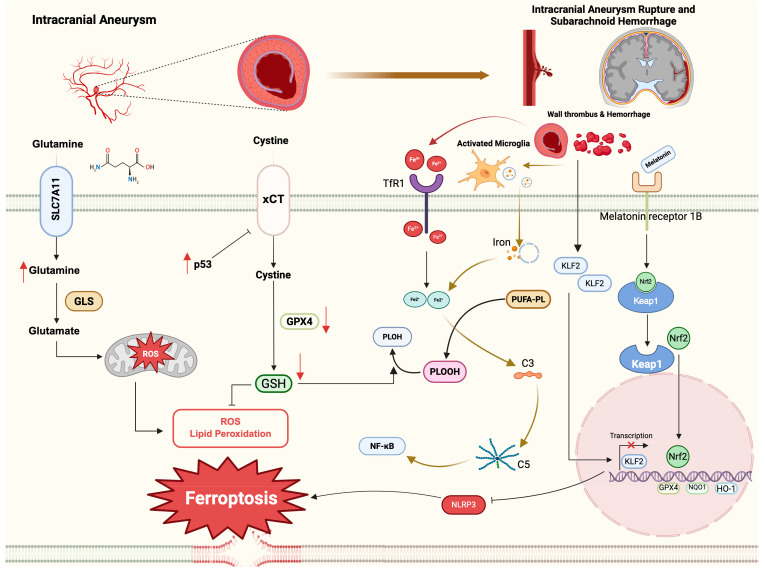
Ferroptosis in intracranial aneurysm and subarachnoid hemorrhage. IA and SAH represent distinct but consecutive stages of cerebrovascular injury. In IA, iron deposition within the aneurysm wall promotes lipid peroxidation via the Fenton reaction, triggering ferroptosis and contributing to vascular wall degeneration and instability. Metabolomic fingerprinting identifies glutamine as a signature metabolite of unstable aneurysms, which fuels ferroptosis through glutamine metabolism and exacerbates aneurysm wall injury, thereby increasing the risk of rupture. Following aneurysmal rupture and SAH, hematoma formation induces neuronal ferroptosis through both direct oxidative damage and indirect inflammatory mechanisms, leading to neurological dysfunction. In addition to Fenton reaction–mediated injury, hematoma components are engulfed by microglia, which subsequently release iron-containing exosomes. Upregulation of p53 after SAH contributes to the exacerbation of neuronal ferroptosis. Uptake of these exosomes by neurons activates the C3/C5/NF-κB signaling pathway, further amplifying neuronal ferroptosis. Notably, neuronal ferroptosis after SAH can be attenuated by melatonin via binding to melatonin receptor 1B (MT2), leading to activation of NRF2 and downstream antioxidant genes, including HO-1 and NQO1.

Despite these advances, there remains a notable lack of well-designed animal models and experimental studies systematically addressing the causal role of ferroptosis in intracranial aneurysm development and rupture. Given the central involvement of glutamine metabolism and its rate-limiting enzyme glutaminase (GLS) in ferroptosis regulation [127,128], whether glutamine contributes to ferroptosis in intracranial aneurysms and whether inhibition of glutamine metabolism may represent a potential therapeutic strategy remains a subject for further investigation (Figure 2).

When an intracranial aneurysm ruptures, blood rapidly extravasates into the subarachnoid space, leading to abrupt and severe clinical manifestations such as intense headache, nausea, vomiting, and, in severe cases, loss of consciousness [129]. Simultaneously, peripheral macrophages are recruited into the subarachnoid space to clear extravasated erythrocytes, during which hemoglobin is degraded and iron is released, a process that closely parallels iron-mediated injury mechanisms observed following intracerebral hemorrhage (ICH) [130]. Iron, a key player in SAH pathogenesis, has been implicated in various aspects of the condition. Studies have shown that the iron-chelating agent deferoxamine effectively reduces SAH mortality, iron-binding protein expression, and neuronal death in rat models of SAH [131]. Additionally, Fe^3+^ has been linked to cerebral microvasospasm, a severe complication of SAH that can be ameliorated by the iron chelator deferoxamine [132]. Cerebral vasospasm restricts intracranial blood vessels, leading to reduced brain blood flow and the accumulation of metabolic by-products and free radicals, triggering oxidative cascades [133,134]. Iron, through the Fenton reaction, further contributes to the generation of harmful hydrogen peroxide and hydroxyl radicals. SAH also activates microglia-involved neuroinflammatory reactions, generating reactive oxygen species (ROS) that induce lipid peroxidation in the brain via cytokines such as IL-6, IL-1β, and TNF-α through the NF-κB pathway [135].

Ferroptosis emerges as a contributor to neural damage following SAH, leading to increased blood–brain barrier permeability, brain edema, and neurological dysfunction [136,137,138]. The ferroptosis inhibitor Lip-1 inhibits microglial amoeboid deformation, release of inflammatory cytokines, and inflammatory reactions [139]. At 24 h post-SAH, brain tissue exhibits decreased GPX4 expression and increased lipid oxidation, with GPX4 overexpression alleviating lipid peroxidation and neuronal death [138]. Elevated iron content and Trf1 expression in brain tissue after SAH are mitigated by the inhibition of Trf1, significantly reducing intracellular iron accumulation and lipid oxidation [137].

Several studies have explored ferroptosis as a target to alleviate secondary nerve injury post-SAH. Downregulating the expression of NCOA4 and increasing intracellular ferritin heavy chain content in brain tissue inhibits S100A8 expression in microglia, alleviating neuronal ferroptosis [140]. Melatonin, upon binding to melatonin receptor 1B, activates NRF2 and downstream antioxidant genes *HO-1/NQO1*, thereby ameliorating neuronal ferroptosis after SAH [141]. Baicalin, a primary component of Scutellaria baicalensis, reduces the accumulation of iron ions and ROS in neurons after SAH, inhibiting neuronal ferroptosis [142]. The selective p53 inhibitor pifithrin improves neurological prognosis, brain edema, alleviates ferroptosis and inflammation in brain tissue, and enhances reduced levels of SLC7A11 and GSH after SAH in animal models [143]. Beyond neuron-intrinsic mechanisms, epigenetic modulation also appears to play a role, as inhibition of KLF2 acetylation mitigates ferroptosis after SAH through suppression of the NLRP1 pathway [144]. Emerging evidence further highlights the contribution of microglia to neuronal ferroptosis, with recent studies demonstrating that microglia-derived exosomes promote neuronal iron accumulation via activation of the C3/C5/NF-κB signaling pathway, thereby triggering ferroptosis after SAH [145] (Figure 2).

Although growing evidence suggests a role for ferroptosis in IA and SAH, several important limitations remain. Direct clinical evidence of ferroptosis in patients is still insufficient. Current IA studies mainly rely on imaging and pathological findings of iron deposition and the expression of COX2, while systematic assessment of lipid peroxidation and other core ferroptosis markers is largely lacking. In SAH, access to human brain tissue is extremely limited because clinical management primarily involves vascular interventions rather than parenchymal resection, with tissue samples available only in rare decompressive surgeries. Consequently, mechanistic insights largely depend on cerebrospinal fluid, blood biomarkers, and experimental models, limiting direct clinical validation. Moreover, the small volume of aneurysmal tissue and the predominance of endovascular treatment further restrict specimen availability in IA. Animal models, including elastase-induced IA and autologous blood–induced SAH models, only partially recapitulate human disease, as IA models fail to reflect the chronic natural history and SAH models predominantly capture acute injury. These limitations highlight the need for clinically relevant approaches that integrate patient-based data to better define the contribution of ferroptosis to IA and SAH.

**Table 1:** Summary of ferroptosis-related mechanisms and clinical implications in intracranial aneurysms.

References	Study Types	Samples	Molecular Mechanism(s)/Impact on Intracranial Aneurysm
Matsushige et al., 2023 [121]	Clinical prospective study	Human IA samples	7T MRI demonstrates microstructural remodeling of giant aneurysm walls, characterized by thrombus organization, repeated intramural hemorrhage, and intramural iron deposition, suggesting iron-driven pathological progression.
Rodemerk et al., 2020 [123]	Clinical prospective study	Human IA samples	Iron deposition within the aneurysm wall is positively correlated with the expression of the ferroptosis marker COX-2; low-intensity MRI signals may serve as a noninvasive indicator of aneurysm wall inflammation.
Li et al., 2022 [124]	Bioinformatics analysis	Four GEO dataset from IA patients	A total of 28 differentially expressed ferroptosis-related genes were identified, leading to the development of a potential intracranial aneurysm diagnostic model incorporating MT3, CDKN1A, ZFP69B, and ABCC1.
Zhu et al., 2022 [125]	Bioinformatics analysis and Clinical pathology	Two GEO datasets from IA patients	Construction of an intracranial aneurysm–associated ferroptosis-related ceRNA network revealed PVT1/*hsa-miR-4644*/SLC39A14 and DUXAP8/*hsa-miR-378e*/378f/SLC2A3 as key regulatory axes
Fang et al., 2025 [146]	Animal study	Human brain vascular smooth muscle cells	KLF15 is involved in the inhibition of vascular smooth muscle cell ferroptosis through interaction with p53 and regulation of SLC7A11 transcription.
Wu et al., 2025 [147]	Clinical prospective observational study	Blood samples from IA patients	Serum ferroptosis markers, characterized by elevated ACSL4 and reduced GPX4 levels, are significantly associated with poor outcomes after intracranial aneurysm rupture and with systemic inflammatory responses.
Ji et al., 2024 [148]	Bioinformatics analysis	single-cell RNA sequencing datasets of both human and murine models	A proinflammatory neutrophil subpopulation (Neu-3) was identified, revealing that neutrophils induce vascular smooth muscle cell ferroptosis via the ALOX5AP/PTGS2 pathway.

## 8. Conclusions and Future Directions

Accumulating evidence indicates that ferroptosis plays a critical role in the pathophysiology of cerebrovascular diseases, including intracerebral hemorrhage, atherosclerosis, ischemic stroke, subarachnoid hemorrhage, and intracranial aneurysms. Dysregulated iron metabolism, excessive lipid peroxidation, and impaired antioxidant defense systems collectively contribute to neuronal and vascular injury, making ferroptosis an attractive therapeutic target. Experimental studies have demonstrated that pharmacological or genetic modulation of ferroptosis-related pathways can alleviate tissue damage and improve neurological outcomes, underscoring its translational potential.

Despite these advances, several challenges remain. The precise molecular mechanisms and cell-type–specific roles of ferroptosis within the neurovascular unit are incompletely understood, and direct clinical evidence remains limited due to restricted access to human brain tissue and the lack of specific ferroptosis biomarkers. Future studies should prioritize the integration of advanced imaging, circulating biomarkers, multi-omics analyses, and clinically relevant models to better characterize ferroptosis dynamics in patients. Moreover, emerging forms of regulated cell death—such as cuproptosis, disulfidptosis, and PANoptosis—may interact with ferroptosis through shared molecular networks, suggesting that cerebrovascular injury is driven by coordinated cell death programs rather than a single pathway.

Given the complexity and heterogeneity of cerebrovascular diseases, combination strategies targeting ferroptosis alongside complementary pathological processes are likely required. Continued mechanistic exploration and rigorous clinical validation will be essential to translate ferroptosis-based interventions into effective therapies, ultimately advancing precision treatment for cerebrovascular disorders.

## Data Availability

No new data were generated or analyzed in this study.
